# Studying the link between physiological performance of *Crotalaria ochroleuca* and the distribution of Ca, P, K and S in seeds with X-ray fluorescence

**DOI:** 10.1371/journal.pone.0222987

**Published:** 2019-09-26

**Authors:** Mayara Fávero Cotrim, Josué Bispo da Silva, Flávia Mendes dos Santos Lourenço, Anielli Verzotto Teixeira, Ricardo Gava, Charline Zaratin Alves, Ana Carina da Silva Candido, Cid Naudi Silva Campos, Márcio Dias Pereira, Salvador Barros Torres, Gianluigi Bacchetta, Paulo Eduardo Teodoro

**Affiliations:** 1 Federal University of Mato Grosso do Sul—UFMS, Chapadão do Sul, MS, Brazil; 2 Federal University of Mato Grosso do Sul—UFMS, Três Lagoas, MS, Brazil; 3 State University of Sao Paulo–UNESP, Ilha Solteira, SP, Brazil; 4 Federal University of Rio Grande do Norte–UFRN, Natal, RN, Brazil; 5 Federal University of the Semi-arid–UFERSA, Mossoró, RN, Brazil; 6 Universitá Degli Studi Di Cagliari, Cagliari—CA, Italy; University of Innsbruck, AUSTRIA

## Abstract

This study describes the use of X-ray fluorescence spectroscopy in *Crotalaria ochroleuca* seed technology. This work evaluated X-ray fluorescence techniques to estimate the physiological performance of different *C*. *ochroleuca* seed coat colours based on the concentration and distribution of Ca, P, K, and S in seed structures. The treatments consisted of seeds separated by coat colours (yellow, green, and red) and a control treatment (colour mix according to their natural occurrence in commercial lots), and was carried out in a completely randomized design, with four replications. The physiological performance was evaluated by analyzing the water content, germination, first germination count, germination speed index, electrical conductivity, seedling emergence, and seedling length and dry mass. X-ray fluorescence spectroscopy techniques were carried out with quantitative analyses (Ca, P, K, and S concentration in the seed coat and the whole seed) and qualitative analyses (macronutrient mapping). The EDXRF and μ-XRF techniques are efficient and promising to differentiate the physiological performance of *C*. *ochroleuca* seeds, based on the concentration and distribution of Ca, P, K, and S in different structures. Ca is predominant in the seed coat, and K, S, and P are found throughout the embryonic axis. Seeds of yellow and green coats have higher nutrients concentration and distribution in the embryonic axis, revealing high germinative capacity and physiological performance. Seeds of red coat have higher nutrients concentration in the seed coat and lower assimilation, showing less vigour, which interferes directly in the quality of commercial lots.

## Introduction

Understanding the seedlings functioning at their early stages is essential to ensure good field establishment. In the first days, seeds absorb few nutrients from the soil, depending exclusively on the reserves stored during the maturation process.

The seed electrical conductivity test has indirect determination of membrane integrity by estimating the release of solutes during the imbibition moment, through K^+^ readings and several salts released in the imbibition solution, soon high levels of leakage are a characteristic of low vigour seed, with low field emergence, particularly in cold, wet soils [1]. A promising alternative to provide faster and more consistent information may be the use of X-ray fluorescence techniques to estimate physiological performance based on the nutrient concentration present.

X-ray fluorescence methods (XRF) are an analytical and microscopic technique used to investigate mineral nutrition, considering that the macronutrient concentration and its location in the seed structures is essential to predict the early physiological performance of seedlings. Elementary maps provide information on several nutrients simultaneously. They also provide practical, useful, and rapid scientific results [2;3]. Phosphorus (P) reserves enable the growth of maize seedlings for two weeks after germination, until the plant has three or more leaves, and promote a vigourous root system in a medium without P [4]. Calcium (Ca) is found at a higher concentration in the seed coat, as verified in soybeans and common bean [5]. In rice seeds, after 48 hours of germination, potassium (K) is highly mobilized and concentrated in the radicle and plumule when the seeds begin to differentiate [6]. The adequate translocation of sulfur (S) to the seed tissues is necessary to maximize and improve the production and quality of proteins [7,8], interfering with seed vigour.

*Crotalaria ochroleuca* L. stands out for its potential of nitrogen fixation [9] and water drought stress tolerance [10]. Moreover, it reduces nematodes populations and promotes soil nutrient cycling [11] and biomass production [12]. Despite the advantages of this legume, the main challenge of *Crotalaria ochroleuca* cultivation is in the low quality of seeds available in the market. The demand for seeds of this plant has increased mainly for its potential to reduce nematodes. The use of quality seeds is fundamental for a uniform and satisfactory establishment of the plants.

The non-uniform flowering and maturation of *C*. *ochroleuca* may lead to heterogeneous seeds regarding chemical composition, seed coat colour, size, germination, and vigour [13,14]. Studies on the chemical composition of the seed coat [15] and compounds stored in the seed are essential for they are responsible for the nutrients distribution to the embryo at the germination stage [16]. The cause of tegument colour variation may be associated with genotype or maturation; however, studies with this species are scarce.

Works on seed technology have focused on the early stages of seedling establishment. They have aimed to identify the causes of variation in germination behavior and verify the chemical composition, which influences storage potential and germination. This study hypothesizes that the colour of *C*. *ochroleuca* seeds affects physiological quality. This work aimed to evaluate X-ray fluorescence techniques to estimate the physiological performance of different *C*. *ochroleuca* seed coat, based on the concentration and distribution of Ca, P, K, and S in the seed structures.

## Materials and methods

*C*. *ochroleuca* seeds from commercial lots were packed in paper bags and stored at 17° C and 50% relative humidity during the experimental period. The treatments consisted of manually separating seeds according to the seed coat colours (yellow, green, and red) and a control treatment (sample with 18% red seed coat, 34% green seed coat, and 48% yellow seed coat, by natural occurrence), in a completely randomized design with four replications. Before the tests, all seeds were washed in sodium hypochlorite solution (0.5% concentration) during 1 minute, so that there was no contamination and interference in the evaluations.

### Physiological performance analyses

The physiological performance analyses consisted of determining the water content, first germination count, germination, germination speed index, electrical conductivity, emergence, and seedlings length and dry mass.

**Water content.** was calculated by the oven method, at 105 ± 3° C, for 24 hours [17], with two sub-samples containing about 2g for each treatment (dry weight basis).

**First count germination and total germination.** Both tests were carried out simultaneously with four replications of 50 seeds per treatment. Seeds were placed in gerbox boxes (11x11x3cm) on blotting paper, moistened with an amount of distilled water equivalent to 2.5 times the mass of the non-moistened paper, and kept for 20°C during at 16h in the dark -30°C for 8 hours of light with 80 Watts total lamps and the evaluations consisted of the percentage of normal seedlings with its well developed shoot and root structures verified on the fourth (first count germination) and tenth days (total germination) after the test installation [17].

**Germination speed index.** Simultaneously to the germination test, seeds with primary root longer than 2 mm were counted, and the index was calculated using the formula: IVG = N1/DQ +N2/D2 + . . . . + Nn/Dn. Where GSP = Germination speed index; N = number of seedlings checked on the day of counting with 2 cm of root and shoot; D = total number of days [18].

**Electrical conductivity.** four replications of 50 seeds had the mass determined in a precision analytical scale (0.0001 g). They were then placed in plastic cups containing 50 mL of distilled water and kept at 25°C. After a 60-minute soak period, the electrical conductivity was measured using a conductivity meter [19].

**Seedlings emergence.** Four sub-samples of 25 seeds were sown in a 128-cell polystyrene-expander tray with one seed per cell, at 1.5 cm depth, containing commercial Maxxi substrate (poultry manure with lime, decomposed processed barks, and expanded vermiculite), kept in a greenhouse with two daily watering. The emerged normal seedlings were counted from the opening of the first pair of leaves, carried out on the tenth day after the test installation.

**Seedlings length and dry mass.** The seedlings considered as normal in the seedlings emergence test were evaluated for hypocotyl length (shoot) and radicle. To determine the dry mass, both parts of the seedlings were separated with a stainless-steel blade, packed in paper bags, and kept in a forced-air circulation oven at 80°C, for 24 hours. Afterward, they were weighed on an analytical scale [20].

### X-ray techniques fluorescence

The analyses of energy dispersive X-ray fluorescence (EDXRF) and micro-X-ray fluorescence (μ-XRF) were carried out at the Nuclear Instrumentation Laboratory at the Center for Nuclear Energy in Agriculture (CENA-USP). The EDXRF allowed determining the Ca, K, S, and P concentration in the seeds (yellow, green, and red), which were separated into two sub-samples (seed coat removed before radicle emergence and whole seeds). In the qualitative analysis, the mapping of nutrient distribution mentioned above was carried out using μ-XRF.

## Quantitative analysis

The two subsamples (seed coat and whole seeds) were dried in a forced air circulation oven at 105 ± 0033°C for 24 hours [17]. Afterward, the subsamples were ground in a low-grade granulometry mill (60 mesh) to obtain 1g, weighed in an analytical scale, and packed in a 6.3 mm sample glass (No. 3577—Spex Ind. Inc., USA), sealed with 5 μm thick polypropylene film (No. 3520—Spex Ind. Inc., USA), so that the samples do not move during an analysis. Then, they were placed in the EDXRF-720 equipment for the concentration of the Ca, K, P, and S (mg kg^-1^).

## Qualitative analysis

The seeds were cut longitudinally, dividing the cotyledons, using a stainless-steel blade. The material was placed in 6.3-mm sample holders (No. 3577—Spex Ind. Inc., USA), sealed with 5-μm thick polypropylene film (No. 3520—Spex Ind. Inc., USA), having the inner side of the seed facing upwards. To verify the intensity of the elements Ca, P, K, and S in the sample, the analysis of μ-XRF (Orbis PC EDAX, USA) was performed using Rh x-Ray tubes configured at 40 kV, electric current of 100 μA, beam of 30 μm per spot, with vacuum and dead-time of about 3%. The matrix consisted of 64 x 50 μm, and the pixels produced were interpolated using Excel and the software Origin Lab version 8.0, 2016. The maps of the nutrients Ca, P, K, and S enabled identifying the location of the elements in the regions of the embryonic axis, seed coat, radicle, cotyledon, and micropyle.

## Statistical analysis

The design was completely randomized, with four replications. Means comparison was carried out by the Tukey´s test at the 5% of probability. All data presented normal distribution by the Shapiro-Wilk test at 5% probability. The canonical variables analysis was used to study the interrelation between the variables of physiological performance and the nutrient concentration in the whole seeds separated by treatment, using the Rbio software [21].

## Results and discussion

### Physiological performance

The water content (WC) ranged between 1.9% (Table 1), which is considered uniform, and is within the tolerable range (<2%) [22], because seeds from the same lot must have similar metabolic activity, a fact evaluated by the WC test. The total germination test (G) identified a high germination capacity in yellow and green seeds, considered superior to the minimum necessary for the commercialization of *C*. *ochroleuca* seeds (75% in Brazil) [23], demonstrating these colours seeds had a high germinative capacity under favorable environmental conditions. Red seeds germinated less represented by the control treatment, interfered in the quality of the whole lot. The decrease in the germination may be due to the lower mobilization of reserves, synthesis of enzymatic activities, or changes in cellular turgor [24].

**Table 1:** Analysis of variance summary for water content (WC), germination (G), first count germination (FCG), germination speed index (GSI) and seedling emergence (E) in different seed coat colours of *C*. *ochroleuca*.

Colour	WC	G	FCG	GSI	E
	%			-	%
Control	9.93	79^1^ b	70 b	33 b	68 b
Yellow	9.64	99 a	80 a	39 a	79 a
Green	9.41	97 a	84 a	39 a	77 a
Red	11.32	55 c	50 c	27 c	8 c
	Mean square				
Colour	-	1246.66*	692.00*	97.67*	3389.42*
Residue	-	39.33	5.00	1.72	1.33
CV (%)	-	8.00	3.15	3.80	1.99

^1^ Means followed by distinct letters in the column differ from each other (Tukey p ≤ 0.05).

* Significant by the F test at the 5% of probability. CV: Coefficient of Variation.

Yellow and green seeds had similar behavior in the tests of first count germination (FCG), germination speed index (GSI), and seedling emergence (E). The low germination capacity was observed in red seeds seedlings, showing their essential structures (shoot and root) deformed or absent. That results may be associated with the deterioration process caused by the delayed harvest, resulting in increased respiration and metabolism of reserves due to field stress conditions. Therefore, data indicate that the yellow and green colours are ideal for the harvest and obtainment of *Crotalaria ochroleuca* in the field, and the classification by the seed coat colour can improve the quality of the commercial lot.

Table 2 shows the results of the hypocotyl length (HL), hypocotyl dry mass (HDM), and electrical conductivity (EC), confirming the interference of the red colour in the control treatment and the high physiological performance of yellow and green seeds. However, the radicle length (RL) and dry mass (RDM) tests were not sensitive to differentiate the physiological behavior between the colours of the integument.

**Table 2:** Hypocotyl length (HL), radicle length (RL), hypocotyl dry mass (HDM), radicle dry mass (RDM), and electrical conductivity (EC) between seed coat colours of *C*. *ochroleuca*.

Colour	HL	RL	HDM	RDM	EC
	cm plant^-1^		g seedling^-1^		(μS cm^-1^ g^-1^)	
Control	2.86^1^ ab	2.59	1.40 bc	1.36	104.44 b
Yellow	3.74 a	2.75	1.50 ab	1.38	47.40 a
Green	3.88 a	2.89	1.51 a	1.44	72.09 a
Red	1.96 b	2.90	1.36 c	1.48	329.32 c
Mean square					
Colour	2.37*	0.06^ns^	0.02*	0.01^ns^	50283.43*
Residue	0.14	0.34	0.01	0.02	126.06
CV (%)	13.34	20.91	2.95	3.46	8.12

^1^ Means followed by distinct letters in the column differ from each other (Tukey p ≤ 0.05).

^ns^ and * correspond to non-significant and significant at the p ≤ 0.05 probability level by the F test, respectively. CV: Coefficient of variation.

Red seeds showed a low capacity for cell membranes reorganization during the soaking process by the EC (Table 2), confirming the low vigour. The green and yellow seeds leached less total exudate and stood out from the other seeds, confirming previous results. This test evaluates the amount of electrolytes released by the seeds during soaking, which is directly related to the integrity of the cell membranes and low seed vigour [25]. Thus, the higher the restoration speed of the membranes, the lower is the amount of leachate released into the external environment and, consequently, the higher is the seed vigour. Therefore, the physiological performance tests revealed high germinative capacity and vigour for yellow and green seeds, and lower physiological behavior for red seeds, interfering with lot quality.

### X-ray fluorescence

**Fig 1:**
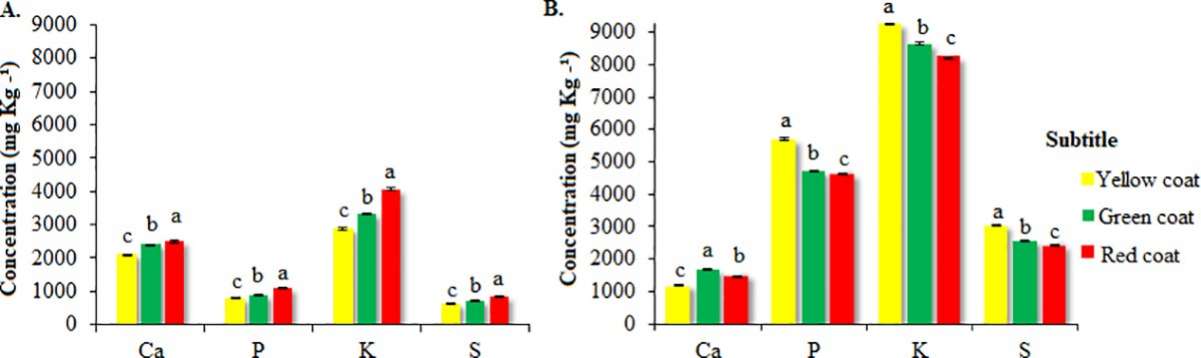
Ca, P, K, and S concentration in the seed coat (A) and whole seed (B) in function of the coat colour of *C*. *ochroleuca* seeds. Bars indicate the standard deviation of the mean (n = 3). Different letters indicate a difference between the means (Tukey, p ≤ 0.05). CVa (%) = 0.79 (Ca), 1.72 (P), 0.96 (K), and 1.39 (S). CVb (%) = 1.25 (Ca), 0.71 (P), 0.22 (K) and 0.61 (S).

**Quantitative results.** The results of conventional bench EDXRF in seed coat and whole seed structures (Fig 1A and 1B) revealed differences among seed colours for nutrient concentration. Red seeds (Fig 1A) showed higher nutrient retention in the seed coat structure when compared with the other colours, with higher Ca and K concentration. Seed coat tissue acts on temporary assimilation higher Ca, P, K and S for subsequent transfer to the embryo, as well as in the protection of internal structures, joint of parts, aid in gas exchange between seed and environment, dormancy and dispersion control, and germination [26]. In addition to these functions, the last ramifications of the phloem are in the coating tissue, which infers the participation of this structure in the translocation of essential elements to the endosperm and embryo [22].

The yellow and green seeds stood out for P, K and S concentration, while the red seeds showed less concentration (Fig 1B). This result shows that the seed coat absorbs more than it retains these nutrients, as confirmed by the P and S concentration in the whole seed. Conversely, the behavior of Ca varied in the different structures, showing higher retention in red seeds and higher concentration in green seeds. The P concentration is an essential agricultural property for this nutrient provides the establishment of vigourous seedlings, increasing the yield [4]. A study on the seed coat of *Arabidopsis thaliana* verified that the P concentration found in this structure at physiological maturity stage is higher than that found in mature and dry seeds. This fact indicates that the previously retained P was redistributed to the embryo, as observed in this study [27].

Therefore, the use of EDXRF allows the efficient estimation of Ca, P, K, and S concentration in seeds with contrasting physiological performance. This tool assesses the concentration of the different essential elements simultaneously and rapidly, ensuring the adoption of procedures such as the treatment of seeds with micronutrients, macronutrients, and biofortification.

**Fig 2:**
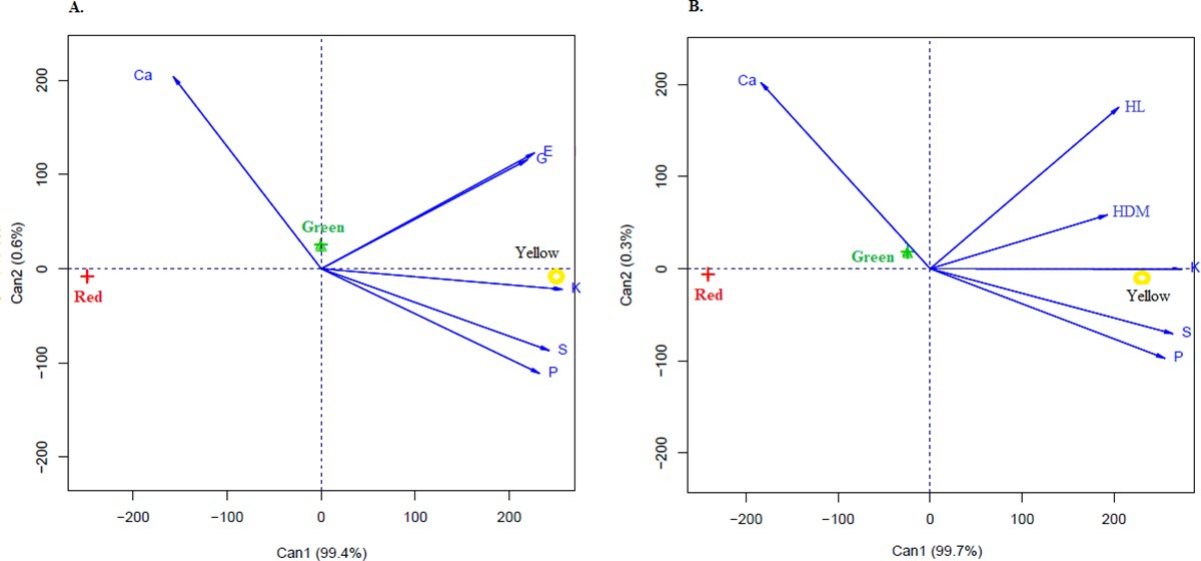
Canonical variables between the tests of germination (G), emergence (E) (A) and hypocotyls length (HL), hypocotyl dry mass (HDM) (B), and Ca, P, K, and S concentration in function of the yellow, green, and red *C*. *ochroleuca* seed coat.

The canonical variables analysis was used to verify the contribution of each variable in the seed coat colour of *C*. *ochroleuca* seeds (Fig 2). For scores to be represented in a two-dimensional graph, the percentage of retained variance must be higher than 80% [28]. In this study, variances accumulated in the two main canonical variables were 99.4 and 99.7%, respectively, for each graph (Fig 2), allowing an accurate interpretation.

The angles (between vectors) smaller than 90° indicate a positive correlation between the macronutrients P, S and K in yellow seeds (Fig 2). Conversely, Ca had a strong correlation with green seeds, showing intermediate performance. Thus, the higher the concentration of the nutrients mentioned above, the higher is the germination (G), emergence (E), and hypocotyls length (HL) and dry mass (HDM) for yellow seeds.

The red seeds were not associated with any of the variables, as shown by the distance of the vectors, confirming the results of the analysis of conventional variance for the physiological performance (Table 1) and nutrient concentration in the whole seeds (Fig 1B). Therefore, the quantification of the nutrient concentration in function of the seed coat colour is fundamental for it directly influences the physiological performance. This procedure improves the processing, storage, and lot quality and enables separating by colours for the adequate establishment of the crop in the field.

**Fig 3:**
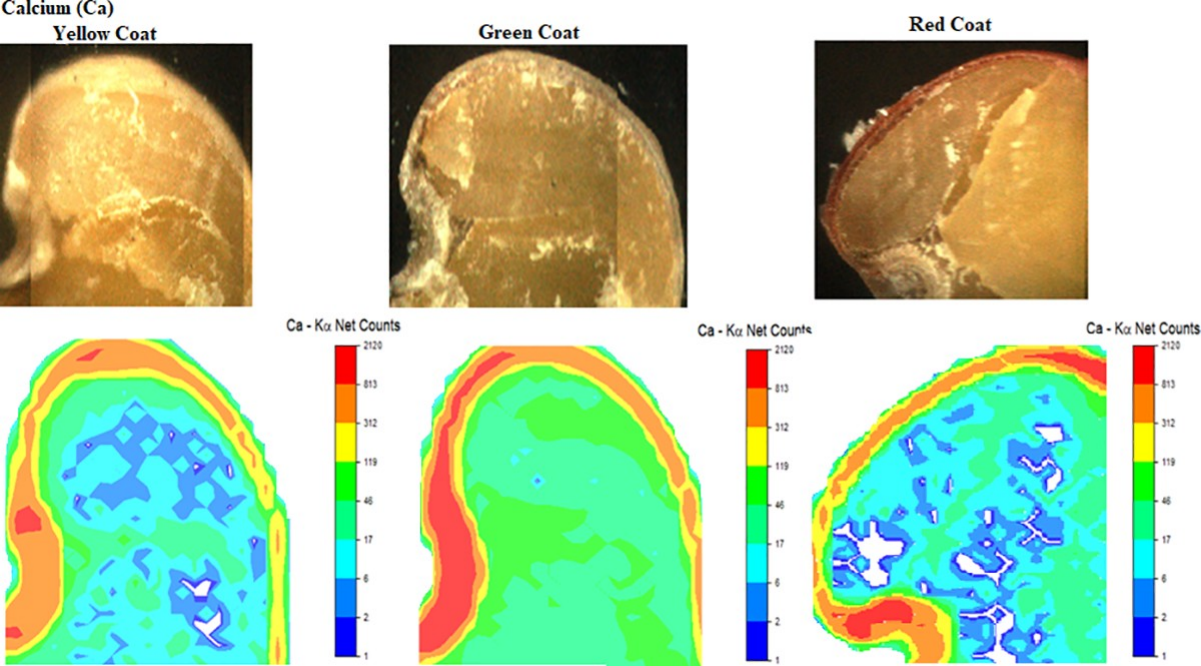
Image captured by μ-XRF and respective calcium distribution map in the region of hypocotyl (H), radicle (R), micropyle (M), cotyledon (C) and yellow (a), green (b), and red (c) seed coat (SC) of *C*. *ochroleuca* seeds.

**Qualitative results.** The analyses of μ-XRF (Fig 3) allowed the precise location of nutrients distribution in each structure of the seed, highlighting the region of the embryonic axis. Ca was verified at a higher intensity in the seed coat (SC) and micropyle (M). Green seeds absorbed more Ca, confirming the analyses of the EDXRF (Figs 1 and 2), as previously shown. A higher intensity of this nutrient is observed in the micropyle region in green seeds. This structure is important in seeds in general since the vascular strand breaks in this area after physiological maturity, and thus represents a region of nutrient discharge. Also, micropyle has an essential connection between maternal tissues [29]. Studies verified that Ca increases the resistance of soybean seed coat to mechanical damage, improving the seed production under field conditions and impairing the entry of pathogens [30].

Calcium ions (Ca^2 +^) in the plasma membrane form the annexins, which are proteins able to interact selectively and non-covalently with phospholipids, found in all eukaryotes. Thus, the primary cell wall of plant cells is composed of cellulose (1 → 4) -β-D-glucan microfibrils, inserted in a matrix of polysaccharides, structural proteins, phenylpropanoids, and a junction zone with Ca [31]. This nutrient acts as pectate in the middle lamella, and as "cementing agents" between nearby cells [32], also serving as an important messenger in the signal transduction of phytohormones, such as the abscisic acid, which acts directly in seedlings germination and growth [6].

Besides being involved in cell wall stiffness by forming pectate complexes, Ca has a signaling role by regulating the cytosol level [33]. In *Vigna radiata* L. seeds, the embryo axis growth at stages I and II of the germination depends on the influx of Ca from the apoplast, and its inhibition is associated with the participation of apoplastic peroxidase, influencing germination and early growth [34].

**Fig 4:**
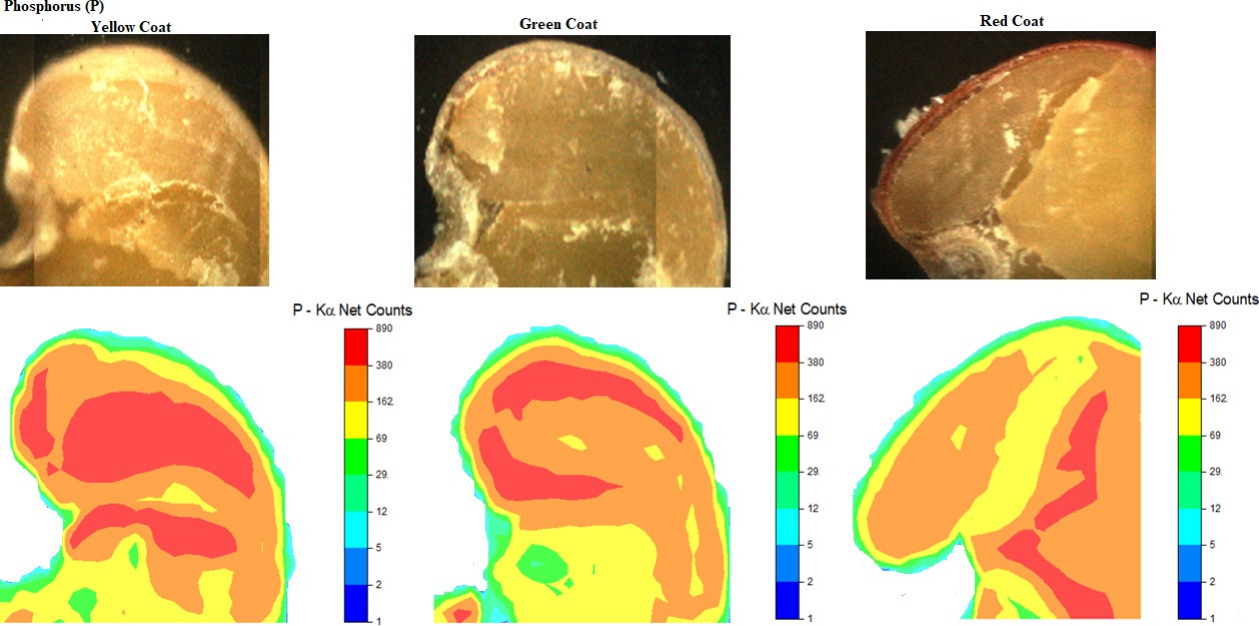
Image captured by μ-XRF and respective phosphorus distribution map in the region of hypocotyl (H), radicle (R), micropyle (M), cotyledon (C) and yellow (a), green (b), and red (c) seed coat (SC) of *C*. *ochroleuca* seeds.

P is distributed throughout the embryonic axis (Fig 4), precisely in the radicle (R) and hypocotyl (H) in yellow and green seeds. However, red seeds exhibited a lower intensity of P in these structures. No expressive P distribution was observed in the coating tissue, corroborating the analyses obtained by the EDXRF (Fig 1). High intensity of this nutrient found in the structures of the radicle, scutellar tissue, and inner layer of the aleurone that surrounds the endosperm and the embryo [35]. The embryo is the most critical structure in the seeds regarding energy saving. This structure is efficient in storing light energy and elements essential for enzymatic functions. It is also essential in the maintenance of the cell turgor [6].

In seeds, about 60 to 80% of the P occurs in the form of phytic acid [36], an important energy source to the metabolic processes at the early stage of germination [37]. This nutrient is vital in several organic compounds, such as nucleic acids, phospholipids, and phosphate esters (including proteins, sugars, and nucleotides) [31].

P is essential to ensure high yield since it acts on cell membranes (phospholipids) and nucleic acids and as energy storage compounds, such as ATP (adenosine triphosphate) [38]. This energy is used at the germination stages, and its deficiency may impact growth regulating mechanisms, as well as decrease enzymes that activate the metabolism of gibberellin. This fact has been verified in seeds of *Arabidopsis thaliana* [39]. The concentration of endogenous P reserves in soybean and maize seeds showed that high nutrient concentrations could produce vigourous seedlings, especially under conditions of element deficiency in the soil [40].

**Fig 5:**
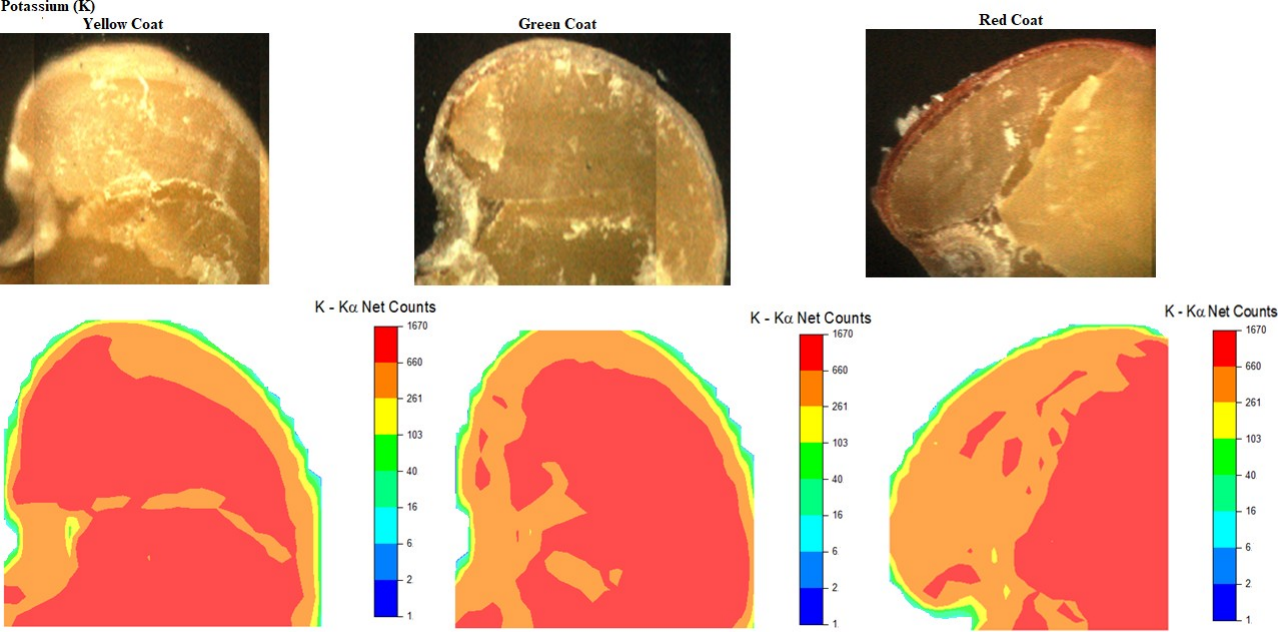
Image captured by μ-XRF and respective potassium (K) distribution map in the region of hypocotyl (H), radicle (R), micropyle (M), cotyledon (C) and yellow (a), green (b), and red (c) seed coat (SC) of *C*. *ochroleuca* seeds.

The structures with an intense presence of K (Fig 5) was the embryonic axis in yellow and green seeds. Red seeds had a lower intensity of K. This nutrient is the inorganic ion present at high concentrations in the seeds, generally exceeding the Calcium (Ca) levels [22]. K has several roles in plant metabolism, such as water absorption control, enzymatic activation, meristematic tissue growth, protein and carbohydrate synthesis, and translocation of assimilates [32]. Also, K acts on electrical neutralization of inorganic and organic anions and macromolecules, pH homeostasis, control of the electric potential of the membrane, and the regulation of the cellular osmotic pressure, with an important function in the movements of cells and organs controlled by the turgor [41].

In rice seeds, after 48 hours of germination, K is highly mobilized and concentrated in the regions of the radicle and plumule, which is when they begin to differentiate [6]. Thus, the growth of the plants from the early stage requires large amounts of K ^+^ ions since during the soaking stage high leaching of salts occurs.

In mature seeds, in general, the balance between the reserve tissue and the embryo is fundamental for the control of seed germination [42]. The reserve tissue detects light signals and interacts with the embryo through bidirectional decomposition during germination [43]. This fact indicates that this structure not only acts as a nodule of nutrients but is also controlled by embryonic signals, assisting the root protrusion and the beginning of germination.

**Fig 6:**
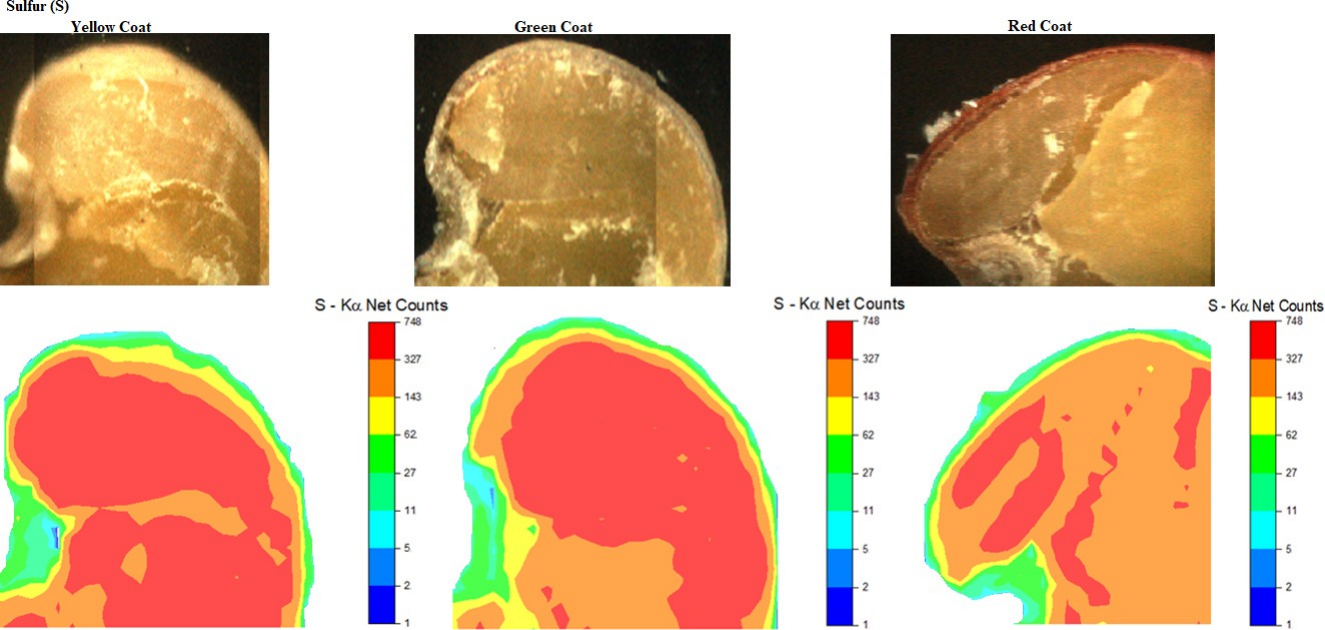
Image captured by μ-XRF and respective sulfur distribution map in the region of the hypocotyl (H), radicle (R), micropyle (M), cotyledon (C) and yellow (a), green (b), and red (c) seed coat (SC) of *C*. *ochroleuca* seeds.

S was observed at its highest intensity along the embryonic axis in yellow and green seeds and, and at its lowest intensity in red seeds (Fig 6). This nutrient acts on fundamental processes, such as electron transport and photosynthetic oxygen production. Thus, sulfur-based defense compounds (elemental sulfur, hydrogen sulfide, glutathione, phytochelatins, S-rich proteins, and other secondary metabolites) are crucial for plant survival during field biotic and abiotic stresses [8].

S is first oxidized to sulfate and then absorbed by plants, acting on the conversion of nitrates to amino acids and then proteins [44,45,46]. Thus, when the levels of S are low, the conversion of amino acids decreases and so does the production of proteins, causing seed quality loss [47]. Therefore, adequate S translocation to seed tissues is necessary to maximize production and improve protein quality [7], directly interfering with vigour.

This research, could infer that high vigour seeds showed sufficient amount of macronutrients to give seedlings with their well defined structures. Seeds with low vigour have lower intensity points in the embryo region and consequently lower nutrient concentration, directly influencing vigour. However, advances in technology may provide complements to current results to assist the understanding of seed physiology in relation to the assimilation and transportation of nutrients during germination. Therefore, future research should be developed to investigate better the role of nutrients (Ca, K, P, and S) in the physiological performance of seeds. The techniques described in this study are promising to differentiate commercial lots with high germinative capacity and vigour.

## Conclusions

EDXRF and μ-XRF techniques are promising to differentiate the physiological performance of *C*. *ochroleuca* seeds based on the levels and distribution of Ca, P, K, and S in different structures. Ca is predominant in the seed coat structure and K, S, and P are found along the embryonic axis, regardless of the seed coat colour.

The yellow and green seed coats have higher assimilation and distribution of nutrients in the embryonic axis, revealing high germinative capacity and physiological performance. In red seeds, the seed coat had higher retention and lower assimilation, showing lower vigour, interfering directly in the quality of commercial lots.

## Supporting information

S1 Data Data file used for statistical analysis presented in Tables 1 and 2, and Figs 1 and 2. (XLS)
